# Recent Trends in Cancer Genomics and Bioinformatics Tools Development

**DOI:** 10.3390/ijms222212146

**Published:** 2021-11-10

**Authors:** Anastasia A. Anashkina, Elena Y. Leberfarb, Yuriy L. Orlov

**Affiliations:** 1 The Digital Health Institute, I.M. Sechenov First Moscow State Medical University of the Ministry of Health of the Russian Federation (Sechenov University), 119991 Moscow, Russia; nastya@eimb.ru; 2 Engelhardt Institute of Molecular Biology, Russian Academy of Sciences, 119991 Moscow, Russia; 3 Department of Medicinal Chemistry, Novosibirsk State Medical University, 630091 Novosibirsk, Russia; leberfarb@mail.ru; 4 Institute of Cytology and Genetics, Siberian Branch of Russian Academy of Sciences, 630090 Novosibirsk, Russia; 5 Life Sciences Department, Novosibirsk State University, 630090 Novosibirsk, Russia; 6 Agrarian and Technological Institute, Peoples’ Friendship University of Russia, 117198 Moscow, Russia

We overview recent research trends in cancer genomics, bioinformatics tools development and medical genetics, based on results discussed in papers collections “Medical Genetics, Genomics and Bioinformatics” (https://www.mdpi.com/journal/ijms/special_issues/Medical_Genetics_2021). This Special Issue continues publications on the “Medical Genetics, Genomics and Bioinformatics” topic presented in *International Journal of Molecular Sciences* from 2019–2020 (https://www.mdpi.com/journal/ijms/special_issues/Medical_Genetics_Bioinformatics) and 2020–2021 (https://www.mdpi.com/journal/ijms/special_issues/Medical_Genetics_Bioinformatics_2), based on the series of medical research conferences held in Russia [1,2].

Molecular mechanisms of cancer progression challenge the development of new bioinformatics tools for diagnostics and systems biology modeling. The presented studies were discussed earlier at the “Systems biology, bioinformatics and biomedicine” symposium in the frames of BGRS-2020 (Bioinformatics of Genome Regulation and Structure) biannual computational biology meeting in Novosibirsk [3,4,5]. The symposium is related to BGRS conference series (https://bgrssb.icgbio.ru/2022/) and followed by the Young Scientists Schools “Systems Biology and Bioinformatics”, which had post-conference journal issues on bioinformatics as well [6,7,8]. The bioinformatics studies focused on molecular mechanisms of gene expression regulation in model organisms were recently published in the *IJMS* Special Issue “Bioinformatics of Gene Regulations and Structure” https://www.mdpi.com/journal/ijms/special_issues/Bioinformatics_Genomics). 

For our overview of recent advances in biomedical frontiers, we acknowledge the medical summit SIBS (Sechenov International Biomedical Summit) organized at I.M. Sechenov First Moscow State Medical University (https://sechenov-sibs.confreg.org/), and other international meetings in Moscow such as “Centenary of human population genetics” [8,9,10] and MGNGS (Medical Genetics—Next-Generation sequencing) (http://ngs.med-gen.ru/) [11]. Over the last two years, many traditional international conferences have been postponed or held online, which has also led to publication delays. The current series of MDPI *IJMS* Special Issues on medical genomics help with communications, and reveal recent trends in medical genomics. Digital medicine technologies, networks and metabolic pathways analysis are on frontiers of computer genomics research.

Overall, the “Medical Genetics, Genomics and Bioinformatics” series collected 28 articles and reviews. This 2021 Special Issue is focused on the analysis of cancer progression [12], genetics mechanisms of cancers [13,14], and bioinformatics theory development [15]. 

The study by Jinmyung Jung and co-authors [12] presents a systems biology approach for molecular network modeling in cancer. The authors explored the conception of genes, which is essential for cancer and specific to a certain mutated gene, using interaction data from the DepMap database (https://depmap.org/portal/). The reconstruction of synthetic partner network (SPN) based on genetic interaction refinement for each mutated gene may help in therapeutic strategies to treat cancer [16]. Thus, this approach extends the analysis of pathways altered in tumor progression.

Extensive usage of the sequencing databases and web-tools, and the integration of bioinformatics methods, form a trend in current medical studies. Cheila Brito et al. [13] used integrated bioinformatics analysis for cutaneous melanoma prognosis. The authors analyzed the expression of ARL (adenosine diphosphate (ADP)-ribosylation factor-like) proteins in melanoma based on extensive data retrieval and expression analysis. The samples were taken from TCGA (the Cancer Genome Atlas ) and GTEx (Genotype tissue expression) resources using Xena project (http://xena.ucsc.edu). Gene expression and immune cell infiltration were determined using the Tumor Immune Estimation Resource (TIMER) server (http://timer.cistrome.org/).

Rueda-Martínez and colleagues [14] studied the genetic contribution to hormone-related cancers development. The authors used genome-wide association studies (GWAS) to evaluate the genetic relationship between endometriosis and hormone-related cancers. The potentially causal associations between endometriosis and cancers were assessed using a two-sample Mendelian randomization (2SMR) approach [17]. 

Alexei Nekrasov et al. [15] presented theoretical work on the analysis of hierarchical organization of protein sequences. The authors developed a modeling method based on the pentapeptide as a unit of protein sequences. This method allows for the revelation of a hierarchical structure in a protein sequence; designing synthetic protein constructs [18]. Thus, this topic on protein structure modeling continues studies by Dmitry Karasev and co-authors [19,20] on the classification of protein sequences presented in “Medical Genetics, Genomics and Bioinformatics” issues. Nurbubu Moldogazieva et al. [21] developed protein–ligand interaction models for estrogen receptor (ERα) and some drugs, thus referring to anticancer therapy strategies.

Integration of bioinformatics data on gene expression, mutagenesis, and pathway interaction analysis form a trend in recent medical genomics studies [12,13]. Immunohistochemistry research in cancer was presented in papers by Anastasiya Snezhkina and colleagues [22,23] in the journal issues of the “Medical Genetics, Genomics and Bioinformatics” series. The authors studied succinate dehydrogenase genes in neuroendocrine tumor—carotid paragangliomas [22]. Mutation analyses of susceptibility genes among patients with head and neck paragangliomas were presented earlier by the same authors group [23]; they revealed *SDHD* gene variants in the majority of the patients. Head and neck paragangliomas can be categorized into carotid body tumors and vagal paraganglioma [24] and classified using TCGA database profiling. Based on exome analysis, Anna Kudryavtseva and co-authors identified pathogenic variants of the *SDHx* genes in vagal paragangliomas [25]. 

Machine learning approaches for personalized oncology also present current trends in medical genomics research [26]. Victor Tkachev and colleagues [27] discussed sequencing data processing in global machine learning methods in clinical oncology. Marianna Zolotovskaia et al. [28] studied molecular heterogeneity of target drugs in oncology.

An important problem in medical genetics is to study associations in mental disorders. It was highlighted in the “Medical Genetics, Genomics and Bioinformatics” Special Issue earlier. Evgeny Ermakov and colleagues [29] analyzed the biochemical mechanisms of schizophrenia. They have linked the disease to immunity and inflammation via immunoglobulins hydrolyzing histones. Chronic low-grade inflammation is associated with schizophrenia. At the same time, extracellular histones may catalyze systemic inflammation that should be prevented. The authors presented the first evidence that polyclonal IgGs of patients, with schizophrenia hydrolyzing the histones. Recently, the same experimental approach of histones hydrolysis was applied to multiple sclerosis study by the authors’ group [30]. Olga Redina et al. [31] studied molecular mechanisms of behavior using laboratory mice models. The authors used RNA-seq of mouse brains areas in behavior experiments. A set of differentially expressed genes was found. The work is based on a bioinformatics analysis of gene expression patterns in animal brain areas [32,33]. Gene network modeling was applied to study aggressive behavior pattern based on associative network reconstruction [34] using ANDSystem (Associative Network Discovery System), a tool for the automatic extraction of knowledge from scientific publications [35]. Ekaterina Trifonova and co-authors used gene network reconstruction to analyze role of mTOR signaling pathway in autism spectrum disorder (ASD) [36,37]. ASD is characterized by high genetic heterogeneity, as well as autoimmune disorders. The authors performed gene-set analysis using SFARI database (https://www.sfari.org/resource/sfari-gene/). Marco Ragusa and colleagues [38] analyzed associations among the alteration of miRNAs and microbiome structure in patients’ saliva, and ASD. The authors studied the association of both miRNAs and microbial environment with ASD scores. Note the gene analysis approach used by Olga Saik and Vadim Klimontov to describe a gene network related to glucose level variability in diabetes [39]. The analysis of gene networks can provide valuable information for a comprehensive understanding of the disease pathogenesis of this disease [40]. The authors employed the ANDSystem based on text mining of scientific publications [35]. The review by Simone Donati and colleagues [41] highlighted the potential role of microRNAs in multiple endocrine neoplasia type 1 syndrome, a rare inherited tumor disease, characterized by the development of multiple neuroendocrine tumors. The deregulation of certain miRNAs species has been associated with this syndrome. 

This Special Issue on medical genomics shows that bioinformatics tools for the analysis of human diseases are in high demand [2,42]. The outputs of disease model analysis come in the form of sets of genes and protein markers which represent a particular interest to medical practitioners [1,2]. “Medical Genetics, Genomics and Bioinformatics” journal issues will be continued in association with BGRS-2022 conference and related symposia (https://bgrssb.icgbio.ru/2022/).
